# Effect of continuous smoking reduction and abstinence on blood pressure and heart rate in smokers switching to electronic cigarettes

**DOI:** 10.1007/s11739-015-1361-y

**Published:** 2016-01-09

**Authors:** Konstantinos Farsalinos, Fabio Cibella, Pasquale Caponnetto, Davide Campagna, Jaymin Bhagwanji Morjaria, Eliana Battaglia, Massimo Caruso, Cristina Russo, Riccardo Polosa

**Affiliations:** Department of Cardiology, Onassis Cardiac Surgery Center, Kallithea, Greece; National Research Council of Italy, Institute of Biomedicine and Molecular Immunology, Palermo, Italy; Centro Per La Prevenzione e Cura Del Tabagismo, Azienda Ospedaliero, Universitaria “Policlinico-V. Emanuele”, Università di Catania, Catania, Italy; Dipartimento di Biomedicina Clinica e Molecolare, Università di Catania, Azienda Ospedaliero, Universitaria “Policlinico-Vittorio Emanuele”, Università di Catania, Catania, Italy; Division of Cardiovascular and Respiratory Studies, Hull York Medical School, Castle Hill Hospital, University of Hull, Cottingham, UK; UOC di Medicina Interna e d’Urgenza, Edificio 4, Piano 3, AOU ‘‘Policlinico-V. Emanuele’’, Via S. Sofia 78, 95123 Catania, Italy

**Keywords:** Smoking cessation, Smoking reduction, Electronic cigarette, Blood pressure, Heart rate, Tobacco harm reduction

## Abstract

We present prospective blood pressure (BP) and hear rate (HR) changes in smokers invited to switch to e-cigarettes in the ECLAT study. BP and HR changes were compared among (1) different study groups (users of high, low, and zero nicotine products) and (2) pooled continuous smoking phenotype classification (same phenotype from week 12 to -52), with participants classified as quitters (completely quit smoking), reducers (≥50 % reduction in smoking consumption) and failures (<50 % or no reduction in smoking consumption). Additionally, the latter comparison was repeated in a subgroup of participants with elevated BP at baseline. No significant changes were observed among study groups for systolic BP, diastolic BP, and HR. In 145 subjects with a continuous smoking phenotype, we observed lower systolic BP at week 52 compared to baseline but no effect of smoking phenotype classification. When the same analysis was repeated in 66 subjects with elevated BP at baseline, a substantial reduction in systolic BP was observed at week 52 compared to baseline (132.4 ± 12.0 vs. 141.2 ± 10.5 mmHg, *p* < 0.001), with a significant effect found for smoking phenotype classification. After adjusting for weight change, gender and age, reduction in systolic BP from baseline at week 52 remains associated significantly with both smoking reduction and smoking abstinence. In conclusion, smokers who reduce or quit smoking by switching to e-cigarettes may lower their systolic BP in the long term, and this reduction is apparent in smokers with elevated BP. The current study adds to the evidence that quitting smoking with the use of e-cigarettes does not lead to higher BP values, and this is independently observed whether e-cigarettes are regularly used or not.

## Introduction

Cigarette smoking is the single most important cause of preventable premature mortality in the world [1]. It is responsible for 50 % of all avoidable deaths in smokers, half of these due to cardiovascular disease [2]. It has been estimated that the 10-year fatal cardiovascular risk is doubled in smokers, while for young smokers the risk for myocardial infarction is up to fivefold higher compared to non-smokers [2, 3, 4]. The risk associated with smoking is primarily related to the amount of tobacco smoked daily, and shows a clear dose–response relationship with no lower limit for deleterious effects [5, 6].

The interaction between smoking and blood pressure (BP) is complex. Smoking causes an immediate elevation of BP and heart rate (HR) due to stimulation of the sympathetic nervous system [7]. However, there is controversy over the independent chronic effect of smoking on BP [8, 9]. In fact, epidemiological studies show that smoking cessation may be associated with an elevated risk for future development of hypertension, which has been attributed to weight gain [10, 11, 12]. In already established hypertension, smoking is associated with an elevated risk for cardiovascular disease; thus quitting smoking is unquestionably among the most important steps patients with elevated BP can take to improve their cardiovascular health [13, 14] [15]. Surprisingly, however, data on the long-term effects of smoking cessation or reduction on BP (and HR) is very limited, and results are unclear, with studies reporting lower, higher or unchanged BP values in smokers compared with non-smokers [16].

Electronic cigarettes (ECs) are an alternative source of nicotine, sharing many similarities with smoking in the behavioural aspect of use [17, 18]. Users are predominantly smokers, who report using the electronic cigarettes long term to reduce cigarette consumption or quit smoking, to relieve tobacco withdrawal symptoms, and to continue having a ‘smoking’ experience but with much reduced health risks [19, 20, 21]. Data from two recent prospective randomised controlled trials show that ECs can aid smoking cessation and reduction [22, 23].

Herein, we present the effects of smoking reduction and abstinence on resting blood pressure (BP) and heart rate (HR) from the ECLAT study—a prospective 12-month double-blind, controlled, randomised clinical three-arm trial designed to evaluate smoking reduction, smoking abstinence and adverse events in apparently healthy smokers not intending to quit after switching to a popular EC brand (‘Categoria’; Arbi Group Srl, Italy). [23] Blood pressure (BP) and heart rate (HR) were compared amongst (1) different study groups (users of high, low, and zero nicotine products) and (2) pooled continuous smoking phenotype classification, with participants classified as quitters (completely quit smoking), reducers (≥50 % reduction in smoking consumption) and failures (<50 % or no reduction in smoking consumption). The latter comparison was repeated in a subgroup of participants with abnormal elevated BP at baseline, to examine the possibility of BP reduction, which would be unlikely to be observed in participants with normal BP at baseline.

## Methods

Details of participants’ characteristics and study design have been previously described [23]. The ethics review board (ERB) of the “Policlinico-Vittorio Emanuele” Hospitals approved the study in June 1, 2010, and participants gave written informed consent prior to participation. The clinicaltrial.gov team subsequently approved the study. The authors confirm that all ongoing and related trials for this drug/intervention are registered. The smokers were recruited during the period June 2010–February 2011 with a final follow-up visit at week 52. The trial registry describes the trial as observational, with a 24-week follow-up, but was conducted as a three-arm RCT with a 52-week follow-up because we decided to monitor the long-term impact of different nicotine levels on smoking cessation or reduction, BP and HR. This is a post hoc analysis, since BP and HR were not officially among the primary or secondary outcomes of trial in the registry entry, but were considered important as safety indicators.

### Participants

Regular smokers not intending to quit were invited to try ECs (“Categoria”, Arbi Group Srl, Italy) as a less harmful alternative to tobacco smoke that could be freely used as a complete substitute for conventional cigarettes. Subjects were made aware that the purpose of the current assessment was to quantify reductions in cigarette consumption by switching to EC use, and the impact on their resting BP and HR on a regular basis at follow-up visits. No financial incentive was offered for participation.

Inclusion criteria were: (1) smoke ≥10 tobacco cigarettes per day (cig/day), for at least the past 5 years, (2) age 18–70 years, (3) in good general health; (4) not currently attempting to quit smoking or wishing to do so in the next 30 days and (5) committed to follow the trial procedures.

Exclusion criteria were: (1) history of cardiovascular disease, respiratory disease, psychiatric disorder or major depression; (2) regular medication use; (3) current or past history of alcohol abuse; (4) use of smokeless tobacco or nicotine replacement therapy, and (5) pregnancy or breastfeeding.

### Study design

**Fig. 1:**
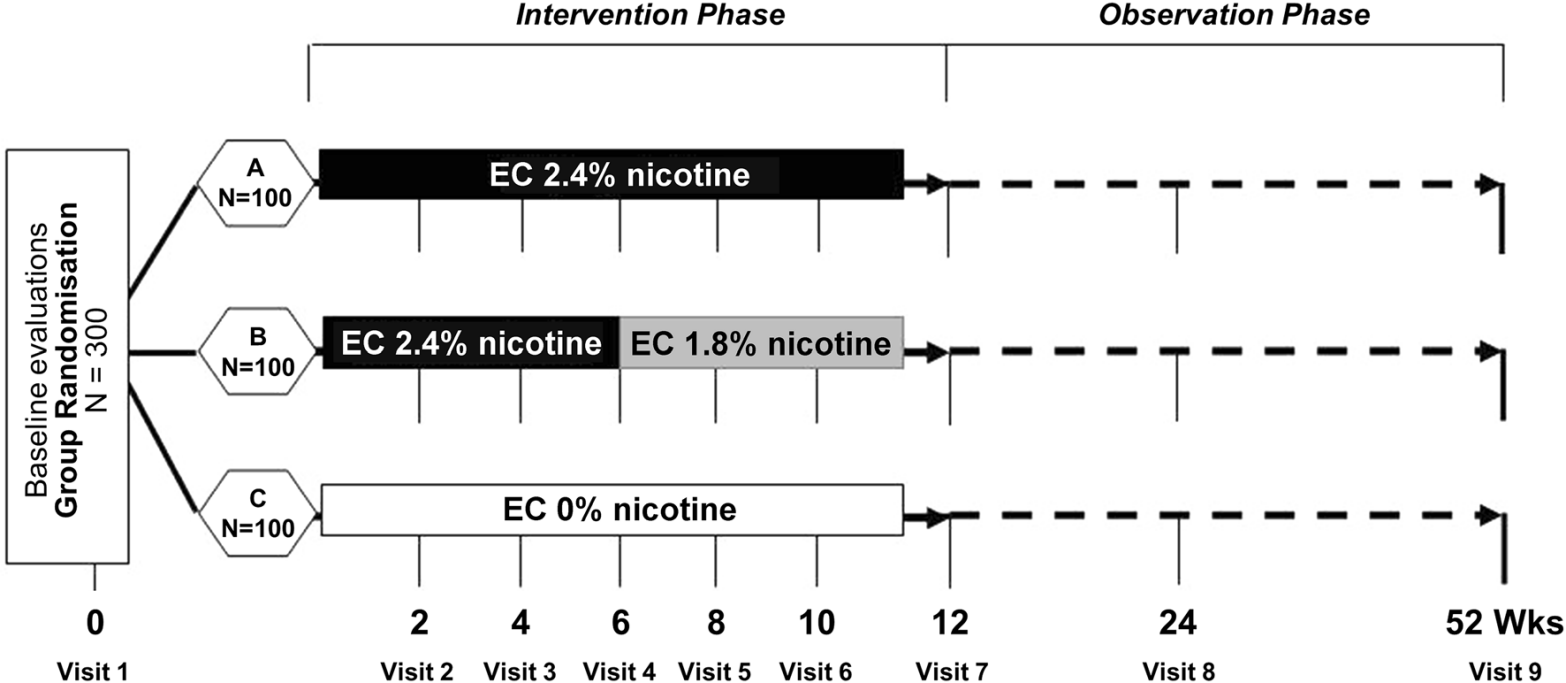
Schematic diagram of the ECLAT study design. Smokers not currently attempting to quit smoking or wishing to do so in the next 30 days were randomised in three study groups: group A (receiving 12 weeks of 2.4 % “Original” nicotine cartridges), group B (receiving 6 weeks of 2.4 % “Original” nicotine cartridges and a further 6 weeks with 1.8 % “Categoria” nicotine cartridges), and group C (receiving 12 weeks of no-nicotine “Original” cartridges). Participants in each group were prospectively reviewed for up to 52 weeks during which smoking habits, eCO levels, BP, HR and body weight were assessed at each study visits

Eligible participants were enrolled in a prospective 12-month randomised, controlled trial consisting of nine office visits at the University Hospital’s smoking cessation clinic (Centro per la Prevenzione e Cura del Tabagismo - CPCT; Università di Catania, Italy). A prospective evaluation of conventional cigarettes consumption, BP and HR was carried out at nine time points (baseline and eight follow-up visits at week 2, 4, 6, 8, 10, 12, 24, and 52). Participants were randomised into three study arms to receive an e-cigarette kit with either “Original” (2.4 % nicotine—Group A), or “Categoria” (1.8 % nicotine—Group B), or “Original” without nicotine (“sweet tobacco” aroma—Group C) cartridges (Fig. 1). The randomization sequence was computer generated, and blinding was ensured by the identical external appearance of the cartridges.

At baseline (visit 1), socio-demographic factors, smoking history, Fagerström Test for cigarette dependence (FTCD) scores and levels of carbon monoxide in exhaled breath—eCO (Micro CO, Micro Medical Ltd, UK) were annotated. Additionally, BP, HR, and body weight were recorded.

Participants were then given a free e-cigarette kit with a full supply of cartridges, and were trained on how to correctly use the product. They were told to use the study product ad libitum (but up to a maximum of four cartridges/day) in the anticipation of reducing cigarette smoking, and to take notes of the daily consumption of conventional cigarettes and cartridge use in their study diaries.

Participants were then invited to return to the CPCT at follow-up visits (visits 2–7) to: (1) receive further free supply of cartridges (with the exception of visit 7) together with the study diaries for the residual study periods, (2) record their eCO levels, (3) have their BP and HR measured, and (4) return completed study diaries and unused study products. At the end of study visit 7, no more cartridges were provided by the investigators, but participants were advised to continue using their EC if they wished to do so. Body weight was also measured at this visit.

Study participants attended two additional follow-up visits at week 24 (visit 8) and at week 52 (visit 9) to report product use and the number of cigarettes per day smoked, and to re-check eCO levels. Resting BP, HR, and body weight were recorded again.

### Office BP and HR measurements

For office systolic and diastolic BP measurements, we followed the methods recommended by the Seventh Report of the Joint National Committee on prevention, detection, evaluation, and treatment of high blood pressure [24]. After a 5-minute rest, BP and HR measurements were obtained by a semi-automated oscillometric sphygmomanometer (Smart Pressure, CA-MI Snc, Parma, Italy). Two measurements in the sitting position, spaced 1–2 min apart, were obtained at each visit. Measurements were taken late in the morning, and participants were asked not to smoke/vape or consume caffeinated drinks for at least 30 min prior to each visit. The average of two measurements was considered for analysis.

### Products tested

The “Categoria” EC (model “401”) was used in this study. It is a three-piece model that closely resembles a conventional cigarette, activated by a rechargeable 3.7 V-90 mAh lithium-ion battery. Disposable cartridges used in this study were of three different types, but of identical appearance: 2.4 % “Original” (2.27 ± 0.13 % nicotine), 1.8 % “Categoria” (1.71 ± 0.09 % nicotine) and “Original” without nicotine (“sweet tobacco” aroma). Detailed toxicology and nicotine content analyses of these cartridges had been carried in a laboratory certified by the Italian Institute of Health and can be found at: http://www.categoriacigarette.com/it/studi-e-ricerche/analisi/analisi-2010. The “Categoria” EC kit and cartridges were provided free of charge by the local distributor, Arbi Group Srl, Italy.

### Smoking phenotypes

Smoking abstinence was defined as complete self-reported abstinence from tobacco smoking (not even a puff) since the previous study visit, which was biochemically verified by eCO levels of ≤7 ppm. Smokers in this category are classified as quitters. Smoking reduction was defined as sustained self-reported ≥50 % reduction in the number of cig/day from baseline (eCO levels were measured to verify smoking status and confirm a reduction compared to baseline) [25]. Smokers in this category are classified as reducers. Smokers who were not categorised in the above categories were classified as failures. The study analysed the effects of BP and HR among continuous smoking phenotypes, which was defined as having the same phenotype from week 12 to 52. Given that long-term changes in BP and HR may become apparent only some time after the change in smoking phenotype, the analysis was performed among participants who had a sustained smoking phenotype for at least 40 weeks.

### Statistical analyses

In our primary analysis, BP and HR values were compared among the study groups (Group A, B, and C: per-protocol analysis). Descriptive data are presented as mean ± standard deviation (SD) or medians and interquartile range (IQ) for normally and not normally distributed variables, respectively. Baseline differences between groups were evaluated by χ^2^ test for categorical variables, and one-way analysis of variance (ANOVA) and Fisher protected LSD for parametric variables; Kruskall–Wallis test was used for non-parametric variables. Repeated measures ANOVA was used to assess changes in systolic BP, diastolic BP and HR from baseline to wek-52, with time as within subject and study group as between subject factors.

In our secondary analysis, BP and HR values were compared among continuous smoking phenotypes, combining datasets from study groups A, B and C (pooled analysis). To evaluate differences at baseline among phenotypes, χ^2^ test, one-way ANOVA, and Fisher’s least significance difference, and Kruskall–Wallis test were used. Repeated measures ANOVA was used to assess changes in systolic BP, diastolic BP and HR, with time (2 time points, baseline and week 52) as within subject and continuous smoking phenotypes (3 phenotypes) as between subject factors. Given that it was improbable (and clinically insignificant) to detect improvements in subjects with normal BP at baseline, the same comparisons were repeated in a subgroup of participants with elevated BP at baseline. These were defined as having high-normal or higher BP values (systolic BP ≥130 mmHg or diastolic BP ≥85 mmHg) [15]. To assess whether continuous smoking phenotypes were associated with changes in BP from baseline to week 52, a linear regression analysis was performed. The difference in BP between baseline and week 52 (ΔBP = week 52−baseline BP) was introduced as dependent variable, and continuous smoking phenotype, age, gender and weight change were introduced as independent factors.

The analyses were carried out using Statistical Package for Social Sciences (SPSS Inc., Chicago, IL) for Windows version 20.0 and two-tailed *p* values of <0.05 were considered significant.

## Results

After screening 417 subjects, a total of 300 (190 males) regular smokers (190 males) were eligible and consented to participate in the study. The baseline characteristics of the participants per study group are presented in Table 1. Baseline characteristics were similar among study groups A, B, and C, with the exception of participants’ age. No difference was observed in systolic BP, diastolic BP, and HR at baseline.

**Table 1:** Baseline characteristics of ECLAT study participants for the overall sample and separately for each treatment arms

	Overall sample (no. = 300)	Group A (no. = 100)	Group B (no. = 100)	Group C (no. = 100)	*P*
Gender (males/females)	190/110	61/39	66/34	63/37	NS
Age (years ± SD)	44.0 ± 12.5	45.9 ± 12.8	43.9 ± 12.2	42.2 ± 12.5	0.040*
Pack years [median (IQR)]	24.9 (14.0–37.0)	24.0 (14.3–37.0)	25.3 (16.9–38.8)	25.5 (12.0–35.0)	NS
Cig/day [median (IQR)]	20.0 (15.0–25.0)	19.0 (14.0–25.0)	21.0 (15.0–26.0)	22.0 (15.0–27.0)	NS
eCO [median (IQR)]	20.0 (15.0–28.0)	19.0 (15.5–29.0)	22.0 (16.0–29.0)	19.5 (14.0–28.0)	NS
FTND (mean ± SD)	5.8 ± 2.2	5.6 ± 2.3	6.0 ± 2.1	5.8 ± 2.2	NS
Past attempts to quit (% yes)	51	56	48	47	NS
Systolic BP (mmHg)	128.0 ± 15.3	127.8 ± 14.2	129.6 ± 17.1	126.7 ± 14.4	NS
Diastolic BP (mmHg)	78.7 ± 10.3	79.6 ± 9.8	78.4 ± 11.4	78.1 ± 9.7	NS
HR (beats per minute)	79.2 ± 1.7	78.2 ± 12.1	80.6 ± 12.7	78.8 ± 10.0	NS
Body weight (kg)	75.0 ± 15.0	74.0 ± 14.2	76.1 ± 15.3	74.8 ± 15.7	NS

Differences among groups were evaluated by χ^2^ test for categorical variables, one-way analysis of variance (ANOVA) and Fisher protected LSD for parametric variables, and Kruskall–Wallis test for non-parametric variables

*SD* standard deviation, *IQR* interquartile range, *cig/day* cigarettes smoked per day, *eCO* exhaled carbon monoxide, *FTCD* Fagerström test of cigarette dependence, *BP* blood pressure, *HR* heart rate

* Difference between groups A and C (one-way ANOVA, Fisher’s least significance difference)

Two hundred and twenty-five subjects (75.0 %) returned at week 12, 211 (70.3 %) at week 24, and 183 (61.0 %) at week 52 for the final follow-up visit. Baseline characteristics of those who were lost to follow-up were not significantly different from participants who completed the study (with the exception of gender; males were 58 % among subjects present at week 52 visit and 71 % among those lost to follow-up, *p* = 0.03), and no significant difference was observed in drop-out rates among study groups at any study visit.

**Fig. 2:**
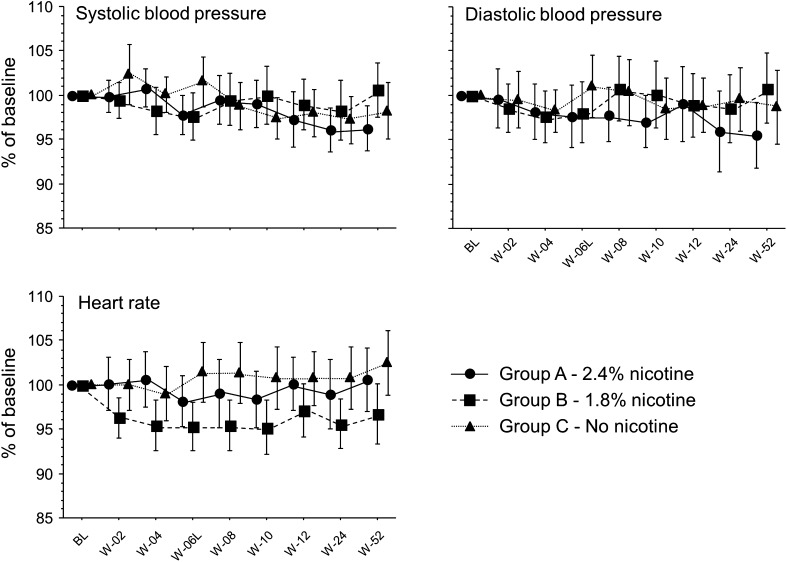
Time course of systolic blood pressure, diastolic blood pressure, and heart rate (in % of baseline) for each step (means and 95 % CI) separately for study groups (A, B, and C). Within subjects changes were significant (*p* = 0.004) only for SBP, while no between subject effect (Group) was found (repeated measures ANOVA)

Overall, reduction and quit rates (%) in the ECLAT study were not significantly different among study groups. In particular, at week 52 the quit rates were 13 % in Group A, 9 % in Group B, and 4 %in Group C. More details about success rates and tolerability with ECs have been reported in the ECLAT study [23]. The time trends of systolic BP, diastolic BP, and HR (in % of baseline value) from all participants that were examined at each follow-up visit are presented in Fig. 2. A slight but significant decrease in systolic BP was found at week 52 (123.1 ± 13.8 mmHg) with respect to baseline (128.0 ± 15.3 mmHg, *p* = 0.004). No significant effect of study groups was observed in any of the parameters.

Among the 183 subjects who completed the follow-up visit at week 52, 145 had a continuous smoking phenotype from week 12 to week 52. The baseline characteristics of these participants are illustrated in Table 2. A small but statistically significant reduction in systolic BP was observed at week 52 compared to baseline (122.6 ± 13.3 vs. 126.0 ± 15.6 mmHg, respectively, *p* = 0.001); no effect of smoking phenotype classification was evident. Also, a small reduction in diastolic BP was observed at week 52 compared to baseline (75.2 ± 9.4 vs. 76.7 ± 9.9 mmHg, respectively, *p* = 0.02). No significant change in HR was observed (81.2 ± 13.0 vs 80.1 ± 11.8 beats/min, *p* = NS).

**Table 2:** Baseline characteristics of ECLAT study participants (*N* = 145) with continuous smoking phenotype classification from week 12 to week 52

	Failures (no. = 93)	Reducers (no. = 34)	Quitters (no. = 18)	*P* value
Gender (M/F)	50/43	22/12	14/4	0.126*
Age (years, mean ± SD)	41.6 ± 13.0	45.4 ± 14.4	44.8 ± 10.5	0.276**
Pack years (median, IQ range)	24.5 (11.1–35.0)	28.3 (15.0–45.0)	23.0 (16.8–33.6)	0.301***
Cig/day (median, IQ range)	20 (15–25)	18 (15–30)	19 (15–20)	0.399***
eCO (median, IQ range)	21 (14–29)	20 (15–26)	17 (12–20)	0.108***
FTND (mean ± SD)	5.9 ± 2.1	5.2 ± 2.1	5.1 ± 2.3	0.182**
Systolic blood pressure (mmHg, mean ± SD)	124.0 ± 15.4	129.4 ± 15.0	130.2 ± 16.9	0.103**
Diastolic blood pressure (mmHg, mean ± SD)	75.8 ± 10.2	77.4 ± 9.7	79.7 ± 7.9	0.281**
Heart rate (beats per min, mean ± SD)	82.3 ± 13.1	79.0 ± 12.5	79.2 ± 13.2	0.350**
Weight (kg, mean ± SD)	70.7 ± 12.5	69.6 ± 12.4	74.4 ± 13.5	0.399**

* χ^2^ test

** One-way analysis of variance (ANOVA) and Fisher protected LSD

*** Kruskall–Wallis test

**Fig. 3:**
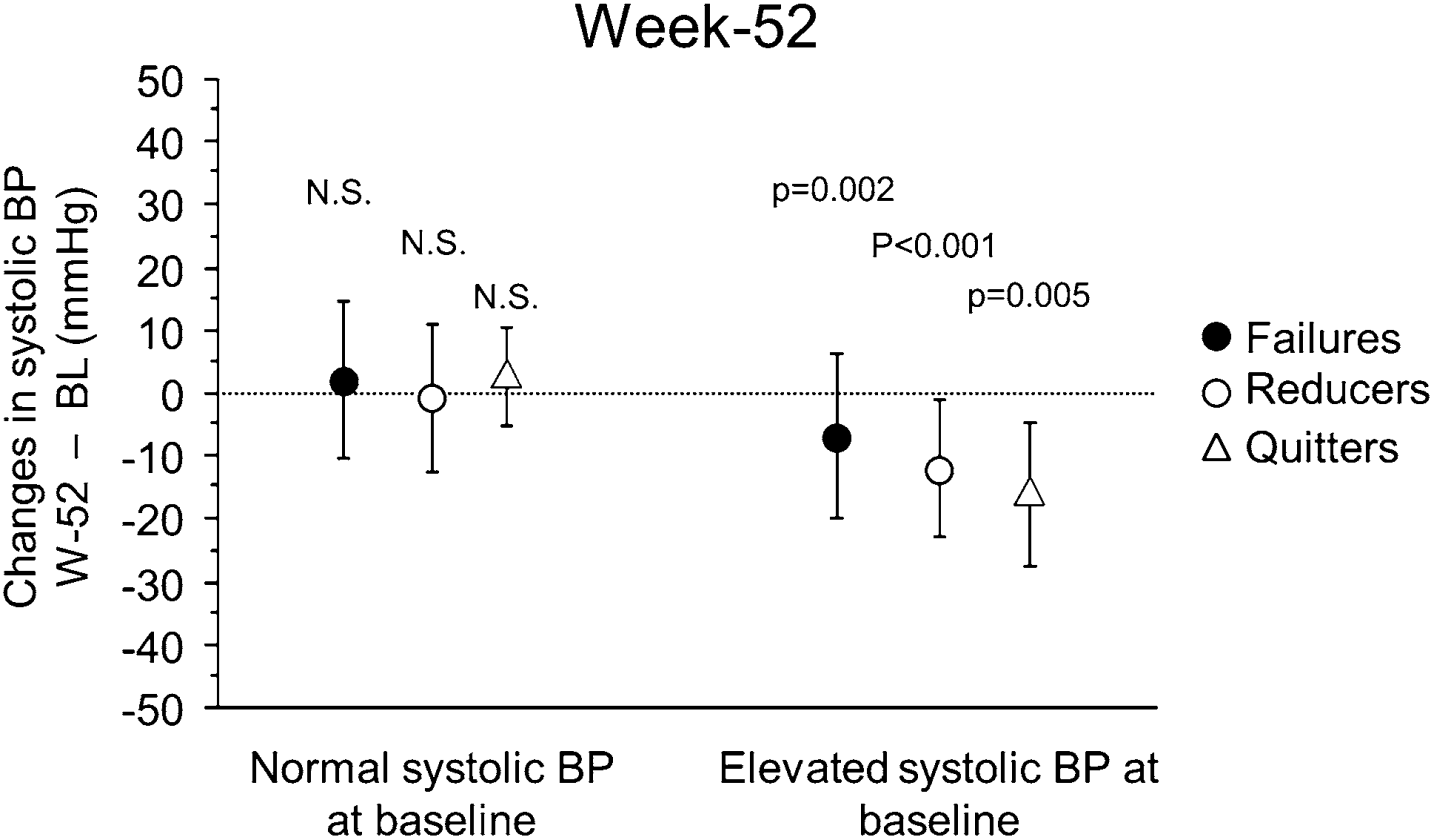
Changes (mean ± SD, absolute mmHg) in systolic blood pressure (SBP) from baseline to week 52 for continuous smoking phenotypes, separately for subjects with normal and elevated SBP at baseline. *P* values for statistical significance of changes from baseline are shown

From the subjects with continuous smoking phenotypes, 66 had elevated BP at baseline. When the above-mentioned analysis was repeated in these subjects, a statistically significant reduction in systolic BP was observed at week 52 compared to baseline (132.4 ± 12.0 vs. 141.2 ± 10.5 mmHg, respectively, *p* < 0.001). A significant effect is found for the continuous smoking phenotype classification, with quitters exhibiting the highest systolic BP reduction (16.3 ± 11.3 mmHg, *p* = 0.005), while Reducers and Failures show reductions of 10.8 ± 10.1 and 6.0 ± 12.5 mmHg, *p* < 0.001 and *p* = 0.002, respectively (Fig. 3). A significant reduction in diastolic BP was also observed at week 52 compared to baseline (77.6 ± 10.2 vs. 82.5 ± 9.8 mmHg, *p* = 0.001). No change in HR is found (79.3 ± 13.5 vs. 82.7 ± 14.5 beats/min, respectively, *p* = NS). No effect of smoking phenotype classification is evident for both diastolic BP and HR. No significant difference in BP changes from baseline is observed in quitters who stop using EC compared to quitters who still use EC (combined for groups A–C).

Of note, changes in body weight from baseline diff among smoking phenotype classifications. Quitters show a small but statistically significant increase in mean body weight from 74.7 ± 12.5 kg at baseline to 75.3 ± 13.5 at week 52 (*p* = 0.038), while no significant changes are observed in reducers or failures. After adjusting for weight change, gender and age, the mean reduction in systolic BP from baseline at week 52 remains associated significantly with both smoking reduction (*p* = 0.046 for reducers) and smoking abstinence (*p* = 0.003 for quitters) (Table 3). The β coefficient for quitters is more that twofold greater in absolute value compared to reducers.

**Table 3:** Multiple linear regression model in which the SBP change from BL to week 52 was entered as dependent variable and tested against continuous smoking phenotype classification, sex, age, and weight change as independent variables

Parameter	β coefficient	95 % CI lower	95 % CI upper	*P* value
Reducers (ref: failures)	−6.76	−13.39	−0.13	0.046
Quitters (ref: failures)	−14.25	−23.70	−4.81	0.003
Sex (female, ref. male)	−4.93	−10.91	1.04	0.106
Age	−0.05	−0.25	0.16	0.659
Weight change (kg)^a^	0.49	−1.38	0.4	0.280

^a^Weight change at week 52 with respect to baseline

## Discussion

This is the first study to investigate long-term changes in BP and HR in smokers who reduce or quit smoking by using ECs in a randomised control trial. Success rates (i.e., ≥50 % smoking reduction from baseline and complete abstinence from tobacco smoking) have been reported in the ECLAT study [23]. Herein, we describe a statistically significant reduction in systolic BP at week 52 in participants with elevated BP at baseline, which is associated with smoking reduction or abstinence even after adjusting for confounding factors. Moreover, similar changes in BP from baseline are observed in quitters who stopped using ECs compared to quitters who still use ECs.

Given the well-established effect of smoking on acute vasopressor and tachycardic responses and increased arterial stiffness, the observed reduction in systolic BP after long-lasting smoking reduction or abstinence is not surprising [26–28]. Nonetheless, the epidemiological evidence is not unequivocal, with some studies showing lower BP values in smokers compared with non-smokers, others reporting no association between smoking habit and blood pressure], and a few others showing that smoking is associated with high BP [29–35]. The current study, which evaluated the effect of a continuous smoking phenotype for 40 weeks (week 12 to 52) on BP, adds to the evidence that quitting does not lead to higher BP values, and this is observed independently of whether ECs are regularly used or not. Population studies have important methodological limitations that may predispose to heterogeneous results. First, these studies rely on self-reported tobacco use and casual collection of BP measurements. Second, because of their cross-sectional design, the observed relationship between levels of smoking and changes in BP does not imply causation. Last but not least, there is the possibility that such studies do not take into account other population characteristics (e.g., age, gender, weight increase, caffeine and alcohol intake), which may play a crucial role when determining potential causation. Moreover, these observational studies were conducted more than 30 years ago, and it is possible that confounders and cut-off limits in particular, might not be valid at the present time. Indeed, the impact of chronic cigarette smoking on BP assessed in a recent cross-sectional study of 33,860 randomly selected adults shows that older male smokers (>45 years old) have d higher systolic (but not diastolic) BP compared to non-smokers when adjusted for age, body mass index, social class, and alcohol intake [9].

Although smoking is not currently considered a risk factor for the development of hypertension, the impact of smoking cessation in patients with elevated or high-normal blood pressure has not been studied adequately (for example, in interventional prospective trials) [15]. In the present randomised controlled trial, a small reduction in BP at week 52 compared to baseline is observed in the whole study population, but no effect of smoking phenotype classification is found. This is not surprising, because it is highly unlikely to detect improvements in smokers with no history of hypertension, and with a normal BP at baseline. Moreover, it is unlikely that any reduction observed in subjects with baseline normal BP is of clinical significance. Although none of the participants was diagnosed as hypertensive, a proportion of them had high-normal or higher BP levels at baseline. In this subgroup of 66 smokers, a more substantial reduction in systolic and diastolic BP at week 52 is observed, with a significant effect now being found for smoking phenotype classification. The findings are important since it is well-established that high-normal BP is a risk factor for future development of hypertension, and is associated with an increased risk of myocardial infarction and coronary artery disease [36, 37]. Mild BP elevations have also been associated with an increased thickness of the carotid media and intima, altered cardiac morphological features and left ventricular diastolic dysfunction [38–40]. Lifestyle changes are recommended in these cases, among which smoking cessation is particularly important. It is, therefore, reassuring that in our smoking cessation study both reducers and quitters have higher reductions in systolic bp compared to failures. the much stronger association observed in quitters, indicates that complete smoking abstinence provides greater benefit compared to smoking reduction.

Of note, the observed reduction in systolic BP remains significantly associated with both smoking reduction and smoking abstinence even after adjusting for age, gender, and weight change in the multiple linear regression analysis. Given the trivial weight gain in quitters at week 52 (only about 0.6 kg), this was not surprising. The observed weight gain is much lower than that reported in the literature [41, 42], despite the fact that quitters were classified based on continuous abstinence over 40 weeks. This suggests that the combination of nicotine delivery and replacement of the rituals associated with smoking behaviour during ECs use might have been the cause for the observed weight gain mitigation in quitters.

In agreement with the findings from other research groups, positive improvements in systolic BP after smoking cessation are noted not only in quitters, but also in reducers [43, 44]. This suggests that the harmful effects of cigarette smoke on the vascular system can potentially be reversed. By substantially reducing exposure to conventional cigarettes’ hazardous toxicants and achieving clinically relevant BP reductions, EC use may not only improve the cardiovascular risk profile but also confer an overall health advantage in smokers unable or unwilling to quit who are also at risk of developing arterial hypertension compared to continuing smoking. The use of low risk nicotine-containing products (including ECs) should be investigated as a safer alternative approach to harm reversal (i.e., specific reversal of BP elevation), and, in general, to harm reduction (i.e., overall reduction of cardiovascular risk associated with tobacco smoking) [45].

Our RCT has the advantage of an interventional prospective trial approach, which minimises the possibility of reverse causality of case–control and cross-sectional studies. Smoking abstinence was biochemically verified at each study visit and BP and HR monitoring was assessed making sure that participants were not smoking/vaping for at least 30 min prior to each measurements. The effects of specific continuous smoking phenotypes were investigated on BP and HR values in the same smokers over several time points for up to 1 year.

There are, however, some limitations. Firstly, participants in this study may represent a self-selected sample (e.g., smokers not intending to quit switching to ECs), which is not representative of all smokers quitting or reducing tobacco smoking. However, it still represents a good cohort of participants to ascertain the effects on BP and HR. Secondly, approximately 40 % of the participants failed to attend their final follow-up visit. Although high attrition rates in smoking cessation studies are not uncommon, this, together with the use of a continuous smoking phenotype classification, and the absence of financial incentive to study participants, might have further contributed to small sample size in some smoking phenotype subgroup cohorts. Thus, results should be interpreted with caution.

Additionally, confounding factors (e.g., salt intake, diet, recreational exercise, alcohol intake) which may have an influence on BP measures were not taken into account. Last but not least, findings from the early first generation e-cigarette (“cigalikes”) under investigation may not be extended to newer-generation devices. It is anticipated that more advanced devices, by allowing a more fulfilling vaping experience compared to “cigalikes”, can be more efficient at reducing or quitting smoking. Whether or not this would indeed have an impact on BP is a separate research question, which requires future testing.

## Conclusions

Smokers who reduce or quit smoking by using ECs may lower their systolic BP in the long term, and this reduction is particularly apparent in smokers with an elevated BP. By showing BP reductions when reducing or stopping smoking for a sufficient period of time, this study adds to the current evidence that EC use appears to be a less harmful alternative to tobacco smoking [46].

In view of the limitation of the previous research applied to this area of clinical science, this paper is likely to set improved methodological approach for future studies addressing the role of smoking cessation and reduction on BP and HR as well as other relevant cardiovascular outcomes. Clinicians are asking for reliable and accurate health information in regular EC users. The evidence-based notion that substitution of conventional cigarettes with ECs is unlikely to raise significant health concerns can improve counselling between physicians and their cardiovascular patients using or intending to use ECs.
